# Changes in Body Water Caused by Sleep Deprivation in Taeeum and Soyang Types in Sasang Medicine: Prospective Intervention Study

**DOI:** 10.1155/2017/2105343

**Published:** 2017-06-06

**Authors:** Seung Min Hong, Byung Joo Kim, Seungwon Shin, Minwoo Hwang

**Affiliations:** ^1^Department of Clinical Korean Medicine, Graduate School, Kyung Hee University, 26 Kyungheedae-ro, Dongdaemun-gu, Seoul 02447, Republic of Korea; ^2^School of Korean Medicine, Pusan National University, 49 Busandaehak-ro, Mulgeum-eup, Yangsan-si, Gyeongsangnam-do 50612, Republic of Korea; ^3^Department of Sasang Constitutional Medicine, College of Korean Medicine, Kyung Hee University, 26 Kyungheedae-ro, Dongdaemun-gu, Seoul 02447, Republic of Korea; ^4^Sasang Medicine Clinic, Kyung Hee University Hospital at Gangdong, 892 Dongnam-ro, Gangdong-gu, Seoul 05278, Republic of Korea

## Abstract

**Background:**

There is a negative relationship between sleep deprivation and health. However, no study has investigated the effect of sleep deprivation on individuals with different body composition. The aim of this study was to determine the differential effect of sleep deprivation in individuals with different body compositions (fluid) according to Soyang type (SY) and Taeeum type (TE).

**Methods:**

Sixty-two cognitively normal, middle-aged people with normal sleep patterns were recruited from the local population. The duration of participants' sleep was restricted to 4 h/day during the intervention phase. To examine the physiological changes brought on by sleep deprivation and recovery, 10 ml of venous blood was obtained.

**Results:**

Total Body Water (TBW) and Extracellular Water (ECW) were significantly different between the groups in the intervention phase. Physiological parameters also varied from the beginning of the resting phase to the end of the experiment. Potassium levels changed more in SY than TE individuals.

**Conclusion:**

Participants responded differently to the same amount of sleep deprivation depending on their Sasang constitution types. This study indicated that SY individuals were more sensitive to sleep deprivation and were slower to recover from the effects of sleep deprivation than TE individuals.

## 1. Background

Sufficient sleep is essential for health and wellbeing. Sleep is closely associated with the regulation of energy balance and metabolism [1], and several studies have shown a relationship between sleep and health [2–5]. Sleep disturbances lead to physical changes; for instance, 48 h sleep deprivation has been reported to increase plasma levels of thyroid hormones [6]. In addition, participants who had been sleep-deprived for 72 h demonstrated increased levels of urea, suggesting increased protein catabolism and gluconeogenesis [7]. Furthermore, disturbed sleep can be lead to disease states. The quantity and quality of sleep has been associated with a greater risk of developing type 2 diabetes [8]. Short sleep duration has also been associated with increased blood pressure and an increased risk of hypertension [9]. Sleeping for less than 6 h or more than 7 h a night is associated with an increased risk of death [2]. However, no study has yet examined the variation in these associations between individuals with different body types.

Sasang constitutional medicine (SCM), developed by Lee Je-ma, is a type of Korean personalized traditional medicine. SCM is widely used for clinical diagnosis and treatment of disease in Korea. SCM classifies people into four types: Taeyang type (TY), Soyang type (SY), Taeeum type (TE), and Soum type (SE) [10]. The Sasang constitution (SC) types have different pathophysiological susceptibilities and have different risks for various diseases [11]. Previous studies have revealed that SC types could be considered risk factors for certain chronic diseases, including diabetes mellitus (DM), hypertension, abdominal obesity, metabolic syndrome, functional dyspepsia, obstructive sleep apnea, and subclinical hypothyroidism [12–15].

SC types are considered to be crucial in predisease and disease stages from a preventive medicine point of view [16]. Therefore, it is also relevant to determine whether SC type is a clinically important risk factor in the predisease stage. However, only one study has found an association between predisease state (pre-HTN) and SC type [11]. Thus, the current study aimed to determine differences in body composition (fluid) in individuals with different SC types after sleep deprivation.

## 2. Methods

### 2.1. Study Design and Intervention

This was a prospective interventional study. The protocol of this study and the consent forms given to participants were approved by the University of KyungHee Institutional Review Board (KHNMCOH 2015-08-002-002). All participants gave written informed consent

**Figure 1:**
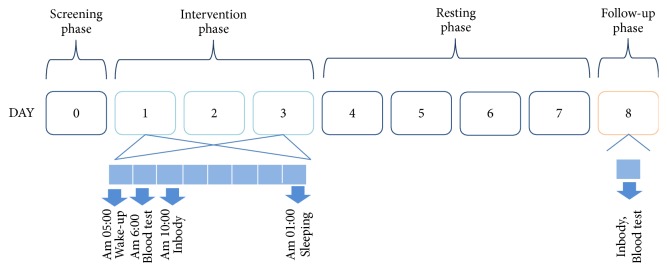
Study design.

As shown in Figure 1, 2 weeks to 1 month after the screening examination, participants registered at the Clinical Research Unit (Sasang Constitution in Kyung Hee University Korean Medicine Hospital in Gangdong (KHUKMHG)) and began the 3-day, 2-night intervention phase of the study (days 1, 2, and 3). During the intervention phase, participants were restricted to 4 h of sleep time and fed a controlled diet based on their weight-maintenance energy by trained research nurses. Participants were asked to avoid caffeine and alcohol for 24 h before the intervention phase. After undergoing sleep restriction (01:00 to 05:00), physiological parameters, including blood test and Inbody test, were assessed. After the intervention phase, participants were allowed to rest for 4 days and 3 nights (days 4, 5, 6, and 7). During the resting phase, participants were allowed to sleep at will. After the resting phase, participants once again visited the Clinical Research Unit and underwent follow-up tests in the follow-up phase (day 8).

### 2.2. Subjects

**Figure 2:**
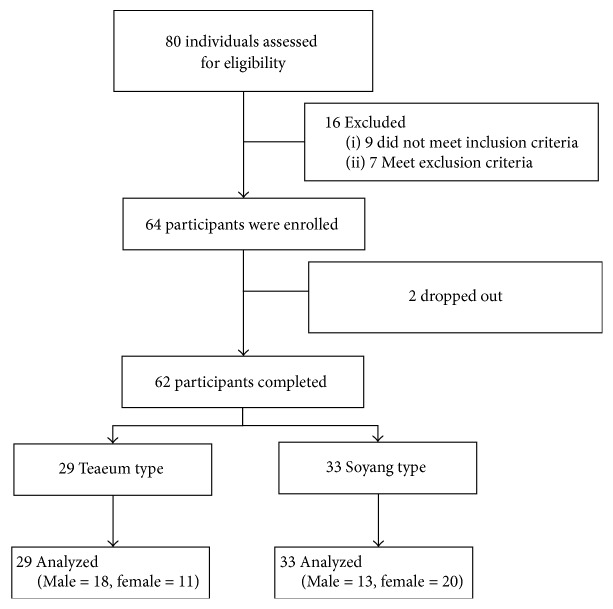
CONSORT flow diagram.

In total, 80 participants (aged 35–45 years) were screened first by interview and then during visits to our clinic. Finally, 62 subjects were included in this study. All subjects met the following criteria: those who had slept 7-8 h per night over the previous week and who did not have sleep disorders, as reflected by a Pittsburgh sleep quality index (PSQI) score under 5 within the last month. Participants who did Chalder Fatigue Scale (CFS) score over 19 within the last month or body pain score as assessed by the Visual Analog Scale (VAS) score 40 mm at any time within the previous month were excluded. Pregnant women or those who has a history of psychiatric or neurological disorders 6 months prior to the study were also excluded (Figure 2).

### 2.3. Data Collection

For the blood test, 10 ml of venous blood was obtained from each subject on four occasions: days 1 and 3 (intervention phase) and day 8 (follow-up phase). During the study, blood was sampled every 30 min starting at 06:00. Blood samples were obtained by peripheral venous puncture, immediately centrifuged at 3000*g* for 10 min and stored at −80°C until required for analysis. Standard methods were used to measure serum electrolyte levels, such as sodium, potassium, calcium, phosphate, creatinine, urea, and uric acid, with an ADVIA 2120i (Siemens, USA).

Participants' body composition was determined with segmental bioelectric impedance using electrodes, according to the manufacturer's instructions (Inbody 770, Biospace Co. Ltd, Seoul, Korea). Daily at 10:00, a pair of electrodes was placed on the surfaces of the thumb, palm, and fingers of the hand, and on the ball of the foot. Microprocessor-controlled switches and an impedance analyzer were used to measure segmental resistance at four frequencies (5, 50, 250, and 500 kHz) Thus, a set of 20 segmental resistances was obtained from each individual. With these data, Total Body Water (TBW), Extracellular Water (ECW), and Intracellular Water (ICW) were calculated from the sum of each segment, using the equations provided in the BIA software [17–19]. The subjects' height and weight were measured and rounded to the nearest hundredth, and body mass index (BMI) was indirectly calculated from the height and weight of each individual.

### 2.4. Diagnosis of SC Type

Diagnosis of the participants' SC types was based on a medical chart review conducted by licensed medical specialists in Korean Sasang typology who had been in clinical practice for 10 years or more. They classified the subjects' SC types based on temperament, body shape, appearance, voice, and pathophysiological characteristics of each individual.

### 2.5. Statistical Analysis

Data obtained before and after the intervention were analyzed using descriptive statistics (Chi-square tests, Mann–Whitney *U* tests, and *t*-tests) based on SPSS software version 17. The level of statistical significance was set at *p* < 0.05. A Kolmogorov-Smirnov test was used to determine whether the data were normally distributed (*p* = 0.9), and the homogeneity of variances was determined (*p* = 0.21).

## 3. Results

### 3.1. Subjects Characteristics

Table 1 shows the general and anthropometric characteristics of the subjects by SC type. The numbers of participants with TE and SY constitutional types were 18 men and 11 women (*n* = 29) and 13 men and 20 women (*n* = 33), respectively. The mean ages of the participants in the TE group and the SY group were 39.86 years and 39.56 years, respectively. Independent* t*-tests revealed that there were no significant differences between the groups in terms of age and height. A chi-square test showed no significant difference between the groups in terms of sex distribution. Overall, both groups had similar background characteristics. Statistical tests also showed no differences between the two groups except for weight and BMI.

### 3.2. Body Composition Changes

Table 2 presents a summary of the body composition of participants measured by Inbody. There was no significant difference between the two groups in terms of TBW, ECW, or ICW during the intervention phase (days 1, 2, and 3) or in the follow-up phase (day 8). However, TBW and ECW varied significantly during the intervention phase (difference between day 1 and day 3, *p* < 0.05), and also during the follow-up phase (difference between day 3 and day 8, *p* < 0.05). No significant difference was found between the two groups in terms of TBW and ECW in a cross-sectional comparison. However, there was a significant difference between TBW and ECW between groups (*p* < 0.05). Variation in TBW during the intervention phase (day 1 to day 3) was 0.14 ± 0.77 in the TE group and 0.51 ± 0.86 in the SY group. Variation in TBW during the resting phase (day 3–8) was 0.34 ± 0.93 and 1.15 ± 0.64 in the two groups, respectively. Variation in ECW during the intervention phase was 0.02 ± 0.28 and 0.18 ± 0.33 in the TE and SY groups, respectively, while variation in ECW during the resting phase was 0.09 ± 0.41 and 0.42 ± 0.31 in the two groups, respectively.

### 3.3. Blood Analysis Changes

Table 3 summarizes the changes in the results of blood analyses. There were no differences in serum electrolyte and albumin levels, or in liver and renal function test results. Electrolyte levels did not change significantly between the intervention phase and the follow-up phase (days 1, 3, and 8). However, potassium level variation showed a constant trend: the variation in potassium levels in the intervention phase (day 3 versus day 1) was 0.09 ± 1.02 and 0.23 ± 0.41 in the TE and SY groups, respectively (Table 3). There was no significant difference between the two groups in terms of liver function and renal function test results in the intervention and follow-up phases (days 1, 3, and 8). Both groups exhibited levels of AST, ALT, BUN, and creatinine that were within normal ranges.

## 4. Discussion

This study focused on SC types as risk factors for the effects of sleep deprivation. There were no significant differences in terms of TBW, ECW, and ICW in the SY and TE groups before the intervention phase. Variation in TBW and ECW was higher in SY individuals than in TE individuals after 3 days of sleep deprivation, whereas variation was lower in TE individuals after 4 days of the resting phase. This indicated that SY-type individuals are more sensitive to sleep deprivation and are slower to recover from physiological changes brought about by sleep deprivation.

TBW was distributed between two compartments, the ECW and the ICW. The composition of ECW is regulated by various mechanisms, but especially by the kidneys [20]. Liver and renal function tests were not significantly different between the two groups. However, potassium levels were different between the two groups. This trend supports the hypothesis that sleep deprivation may influence sodium-potassium pumps. Previous studies have suggested that sleep deprivation increases sodium-potassium pump activity and that this increase may be mediated by the action of norepinephrine on either alpha-1/-2 receptors or increased turnover of sodium-potassium pump molecules [21, 22]. Furthermore, this increased activity of the sodium-potassium pump induced oxidative stress [23, 24]. Therefore, reactive oxygen species (ROS) may be produced during the sleep deprivation process, as suggested by other studies [25, 26].

Metabolic syndrome, a collection of cardiometabolic risk factors that includes obesity, insulin resistance, hypertension, and dyslipidemia [27], is often characterized by oxidative stress, a condition involving an imbalance between the production and inactivation of ROS. Therefore in the present study, although the intervention phase involved only a 3-day, 2-night period, SY individuals may be more susceptible to metabolic syndrome. In addition, the effect of hormones (aldosterone, cortisol, etc.) and the interaction between cardiac output and peripheral arterial resistance may be involved in this difference in individual responses [28–30]. In future, the association between metabolic syndrome, various hormonal changes and cardiovascular diseases, and sleep deprivation and cell volume regulation should be investigated.

According to Sasang constitutional medicine, sleep deprivation is one of a list of symptoms [31] that are used to identify an individual's constitution and a number of constitutional diseases [32]. In fact, a previous study of 1229 patients who attended department of Sasang constitutional medicine for their first medical examination showed that SY individuals typically experienced poor sleep quality and quantity [33].

The main strength of this study is the restriction of sleep deprivation to clinical research conditions. However, there are some limitations to this study. First, the intervention phase was only 3 days, which is too short a period to elicit changes in the body. Second, constitution types were not evenly distributed across study participants but were biased toward the TE and SY. Therefore, the present findings can not be generalized to So Eum type (SE). Future studies should include a large number of participants and should be performed across institutes using a prospective clinical research design, in order to derive a plausible model for predicting disease caused by lack of sleep.

## 5. Conclusion

This study showed that TBW, ECW, and ICW are not significantly different between the SY and TE in a cross-sectional comparison. However, variations in TBW and ECW were greater in SY individuals and were higher than in TE-type individuals after 3 days of sleep deprivation, and SY-type individuals recovered more slowly than TE-type individuals did during the 4-day resting period after sleep deprivation. Liver and renal function parameters and blood sodium, potassium, and albumin concentrations were not significantly different between the two groups, although potassium levels were altered differentially across the two groups. Thus, individuals respond differently to the same amount of sleep deprivation according to SC types.

## Figures and Tables

**Table 1:** Overview of baseline characteristics.

Variable	Taeeum type (*N* = 29)	Soyang type (*N* = 33)	*p* value^*∗*^
	Mean (SD)	Mean (SD)	
Gender, *N*	Male-18	Male-13	0.52
	Female-11	Female-20	
Age (y)	39.86 ± 2.48	39.56 ± 2.83	0.91
Height (cm)	169.84 ± 9.06	166.64 ± 7.43	0.13
Weight (kg)	67.00 ± 10.12	60.02 ± 8.97	0.05^†^
BMI (kg/m^2^)	23.04 ± 1.82	21.49 ± 2.15	0.03^†^
PSQI	3.95 ± 1.02	4.01 ± 1.32	0.45

SD: standard deviation, *N*: number, y: year, BMI: body mass index, and PSQI: Pittsburg Sleep Quality Index.

^*∗*^ *p* values are addressed by Student's *t*-test, *χ*^2^-test between Taeeum type and Soyang type (*α* = 0.05).

^†^ *p* < 0.05 from Student's *t*-test.

**Table 2:** The difference of body water between Taeeum type and Soyang type.

Variable	Taeeum type (*N* = 29)	Soyang type (*N* = 33)	*p* value^*∗*^
	Mean ± SD	Mean ± SD	
ICW (L)			
D3-D1	0.13 ± 0.49	0.37 ± 0.56	0.08
D3-D8	0.23 ± 0.54	0.59 ± 0.88	0.06

ECW (L)			
D3-D1	0.02 ± 0.28	0.18 ± 0.33	0.05^†^
D3-D8	0.09 ± 0.41	0.42 ± 0.31	0.00^†^

TBW (L)			
D3-D1	0.14 ± 0.77	0.51 ± 0.86	0.05^†^
D3-D8	0.34 ± 0.93	1.15 ± 0.64	0.00^†^

*N*: number, SD: standard deviation, ICW: intracellular water, ECW: extracellular water, TBW: total body water, D1: day 1, D3: day 3, D8: day 8, D3-D1: differences in the intervention phase, and D3-D8: differences in the resting phase.

^*∗*^ *p* values are addressed by Student's *t*-test (*α* = 0.05).

^†^ *p* < 0.05 from Student's *t*-test.

**Table 3:** The difference of electrolyte, liver, and renal functions between Taeeum type and Soyang type.

Variable	Taeeum type (*N* = 29)	Soyang type (*N* = 33)	*p* value^*∗*^
	Mean ± SD	Mean ± SD	
K (mmol/L)			
D3-D1	0.09 ± 1.02	0.23 ± 0.41	0.05^†^
D3-D8	1.02 ± 7.04	−0.39 ± 0.54	0.25

Na (mmol/L)			
D3-D1	0.13 ± 1.42	0.00 ± 1.56	0.72
D3-D8	0.27 ± 1.38	0.42 ± 1.63	0.70

Ca (mg/dL)			
D3-D1	−2.31 ± 0.17	−0.15 ± 0.33	0.30
D3-D8	−0.035 ± 0.30	−0.30 ± 0.32	0.51

Alb (g/dL)			
D3-D1	−0.20 ± 0.15	−0.18 ± 0.20	0.65
D3-D8	−0.34 ± 0.22	−0.37 ± 0.19	0.31

BUN (mg/dL)			
D3-D1	−1.33 ± 2.62	−2.19 ± 2.37	0.18
D3-D8	−1.43 ± 3.63	−2.05 ± 2.72	0.45

Cr (mg/dL)			
D3-D1	0.01 ± 0.68	0.00 ± 0.04	0.20
D3-D8	0.00 ± 0.06	−0.01 ± 0.05	0.21

AST (U/L)			
D3-D1	−0.62 ± 3.83	−0.52 ± 5.67	0.93
D3-D8	0.03 ± 4.06	−1.06 ± 6.87	0.46

ALT (U/L)			
D3-D1	−2.34 ± 4.84	−0.18 ± 8.51	0.23
D3-D8	−1.31 ± 4.89	−1.42 ± 8.73	0.95

*N*: number, SD: standard deviation, K: potassium, Na: natrium, Ca: calcium, Alb: albumin, BUN: blood urea nitrogen, Cr: creatinine, AST: aspartate aminotransferase, ALT: alanine aminotransferase, D1: day 1, D3: day 3, D8: day 8, D3-D1: differences in the intervention phase, and D3-D8: differences in the resting phase.

^*∗*^ *p* values are addressed by Student's *t*-test (*α* = 0.05).

^†^ *p* < 0.05 from Student's *t*-test.

## References

[1] Siegel J. M. (2005). Clues to the functions of mammalian sleep. *Nature*.

[2] Patel S. R., Ayas N. T., Malhotra M. R. (2004). A prospective study of sleep duration and mortality risk in women. *Sleep*.

[3] Everson C. A., Szabo A. (2009). Recurrent restriction of sleep and inadequate recuperation induce both adaptive changes and pathological outcomes. *American Journal of Physiology—Regulatory Integrative and Comparative Physiology*.

[4] Kripke D. F., Garfinkel L., Wingard D. L., Klauber M. R., Marler M. R. (2002). Mortality associated with sleep duration and insomnia. *Archives of General Psychiatry*.

[5] Tamakoshi A., Ohno Y. (2004). Self-reported sleep duration as a predictor of all-cause mortality: results from the JACC Study, Japan. *Sleep*.

[6] Feng H.-W., Jiang T., Zhang H.-P. (2015). Comparisons of thyroid hormone, intelligence, attention, and quality of life in children with obstructive sleep apnea hypopnea syndrome before and after endoscopic adenoidectomy. *BioMed Research International*.

[7] Kant G. J., Genser S. G., Thorne D. R., Pfalser J. L., Mougey E. H. (1984). Effects of 72 hour sleep deprivation on urinary cortisol and indices of metabolism. *Sleep*.

[8] Cappuccio F. P., D'Elia L., Strazzullo P., Miller M. A. (2010). Quantity and quality of sleep and incidence of type 2 diabetes: a systematic review and meta-analysis. *Diabetes Care*.

[9] Larcher S., Benhamou P., Pépin J., Borel A. (2015). Sleep habits and diabetes. *Diabetes & Metabolism*.

[10] Kim Y. J., Ahn Y. C., Son C. G. (2015). Sasang constitution affects the prevalence of functional dyspepsia. *BMC Complementary and Alternative Medicine*.

[11] Jang E., Baek Y., Kim Y., Park K., Lee S. (2015). Sasang constitution may act as a risk factor for prehypertension. *BMC Complementary and Alternative Medicine*.

[12] Lee T.-G., Koh B., Lee S. (2009). Sasang constitution as a risk factor for diabetes mellitus: a cross-sectional study. *Evidence-Based Complementary and Alternative Medicine*.

[13] Choi K., Lee J., Yoo J., Lee E., Koh B., Lee J. (2011). Sasang constitutional types can act as a risk factor for insulin resistance. *Diabetes Research and Clinical Practice*.

[14] Jang E., Baek Y., Park K., Lee S. (2013). Could the Sasang constitution itself be a risk factor of abdominal obesity?. *BMC Complementary and Alternative Medicine*.

[15] Song K. H., Yu S.-G., Kim J. Y. (2012). Prevalence of metabolic syndrome according to sasang constitutional medicine in Korean subjects. *Evidence-Based Complementary and Alternative Medicine*.

[16] Do J.-H., Jang E., Ku B., Jang J.-S., Kim H., Kim J. Y. (2012). Development of an integrated Sasang constitution diagnosis method using face, body shape, voice, and questionnaire information. *BMC Complementary and Alternative Medicine*.

[17] Okamoto M., Fukui M., Kurusu A. (2006). Usefulness of a body composition analyzer, inbody 2.0, in chronic hemodialysis patients. *Kaohsiung Journal of Medical Sciences*.

[18] Cha K., Chertow G. M., Gonzalez J., Lazarus J. M., Wilmore D. W. (1995). Multifrequency bioelectrical impedance estimates the distribution of body water. *Journal of Applied Physiology*.

[19] Lee S. W., Song J. H., Kim G. A., Lee K. J., Kim M.-J. (2001). Assessment of total body water from anthropometry-based equations using bioelectrical impedance as reference in Korean adult control and haemodialysis subjects. *Nephrology Dialysis Transplantation*.

[20] Choi H. Y., Ha S. K. (2013). Potassium balances in maintenance hemodialysis. *Electrolyte and Blood Pressure*.

[21] Gulyani S., Mallick B. N. (1995). Possible mechanism of rapid eye movement sleep deprivation induced increase in N-K ATPase activity. *Neuroscience*.

[22] Majumdar S., Faisal M., Madan V., Mallick B. N. (2003). Increased turnover of Na-K ATPase molecules in rat brain after rapid eye movement sleep deprivation. *Journal of Neuroscience Research*.

[23] Liu C.-C., Fry N. A. S., Hamilton E. J. (2013). Redox-dependent regulation of the Na+-K+ pump: new twists to an old target for treatment of heart failure. *Journal of Molecular and Cellular Cardiology*.

[24] Figtree G. A., Keyvan Karimi G., Liu C., Rasmussen H. H. (2012). Oxidative regulation of the Na+-K+ pump in the cardiovascular system. *Free Radical Biology and Medicine*.

[25] Villafuerte G., Miguel-Puga A., Murillo Rodríguez E., Machado S., Manjarrez E., Arias-Carrión O. (2015). Sleep deprivation and oxidative stress in animal models: a systematic review. *Oxidative Medicine and Cellular Longevity*.

[26] Noguti J., Andersen M. L., Cirelli C., Ribeiro D. A. (2013). Oxidative stress, cancer, and sleep deprivation: is there a logical link in this association?. *Sleep and Breathing*.

[27] Roberts C. K., Sindhu K. K. (2009). Oxidative stress and metabolic syndrome. *Life Sciences*.

[28] Cohen J. J., Hulter H. N., Smithline N., Melby J. C., Schwartz W. B. (1976). The critical role of the adrenal gland in the renal regulation of acid base equilibrium during chronic hypotonic expansion: evidence that chronic hyponatremia is a potent stimulus to aldosterone secretion. *Journal of Clinical Investigation*.

[29] Howard R. L., Schrier R. W. (1990). A unifying hypothesis of sodium and water regulation in health and disease. *Hormone Research in Paediatrics*.

[30] Lin S.-H., Halperin M. L. (2007). Hypokalemia: a practical approach to diagnosis and its genetic basis. *Current Medicinal Chemistry*.

[31] Song I. B., KIM S. M. (2000). A Study of ordinary symtoms in the Dongyi Soose Bowon Sasang Chobongyun and DongmuYugo. *Journal of Sasang Constitute Med*.

[32] Choi J. Y., Choi Lee Y. S., Park S. S. (2004). A study on the clinical features of oridinary sleeping patterns based on the sasang constitution, using the logistic regression. *Journal of Korean Oriental Med*.

[33] Kim J. J., Lee Y. S., Park S. S. (2005). A clinical study on the ordinary sleeping patterns of soyangin. *Journal of Oriental Neuropsychiatry*.

